# Small but mighty: targeted antifungal liposomes of a smaller size are superior in treating cryptococcal meningitis

**DOI:** 10.1128/mbio.02507-24

**Published:** 2024-11-18

**Authors:** Tuyetnhu Pham, Peter Zhang, Suresh Ambati, Richard B. Meagher, Xiaorong Lin

**Affiliations:** 1 Department of Plant Biology, University of Georgia, Athens, Georgia, USA; 2 FormuMax Scientific, Inc., Sunnyvale, California, USA; 3 Department of Genetics, University of Georgia, Athens, Georgia, USA; 4 Department of Microbiology, University of Georgia, Athens, Georgia, USA; Albert Einstein College of Medicine, Bronx, New York, USA

**Keywords:** targeted drug delivery, liposome, dectins, *Cryptococcus neoformans*, amphotericin B, cryptococcal meningoencephalitis

## Abstract

Cryptococcal meningoencephalitis (CME) is deadly. CME is responsible for 19% of deaths in AIDS patients, and its global mortality is greater than 60%. The recommended CME therapy requires amphotericin B (AmB), a fungicidal drug targeting fungal ergosterol. AmB also binds to the host’s cholesterol and is highly toxic. Liposomal AmB (AmB-LLs), relative to deoxycholate-solubilized AmB, has lower toxicity and longer tissue retention, but it requires high doses for treatment and its efficacy in treating CME remains unsatisfactory. To improve the effectiveness of AmB-LLs, we previously developed DectiSomes-targeted AmB-LLs decorated with host dectins that recognize fungal polysaccharides. DectiSomes, relative to untargeted AmB-LLs, modestly improve efficacy against systemic cryptococcosis, in contrast to the drastic improvement observed in candidiasis or aspergillosis models. We speculated that limited tissue penetration of the regular-sized DectiSomes might have contributed to the modest improvement in treating systemic cryptococcosis. Here, we discovered that DectiSomes of a smaller size (~50 nm), compared with DectiSomes of the regular size (~100 nm) or untargeted AmB-LLs of either size, had a much better capability in reducing cryptococcal burden of various organs including the brain and in prolonging the survival of mice with systemic cryptococcosis. The performance of small DectiSomes was far superior to all other groups at two different doses of AmB tested. Furthermore, no kidney toxicity was observed in any of the treatment regimens tested. Taken together, our findings indicate that small DectiSomes can be a powerful antifungal delivery platform to drastically improve therapies against the deadly CME.

**IMPORTANCE:**

Systemic cryptococcosis is fatal even with antifungal interventions. The most effective drug against this disease is amphotericin B (AmB). However, AmB is highly toxic as it binds to fungal ergosterol and also mammalian cholesterol. Liposomal AmB was introduced to the clinic in 1990s because it showed reduced toxicity and longer retention in various organs. However, the dose of AmB required for treatment using liposomal formulation is high and the outcome is far from satisfactory. In our previous work, we generated DectiSomes, dectin-decorated liposomes loaded with AmB that more effectively deliver the drug to the pathogen and enhance antifungal efficacy. However, the improvement in treating systemic cryptococcosis, compared with candidiasis and aspergillosis, is modest. Here, we generated DectiSomes that are half their regular size to improve tissue penetration. We discovered that small DectiSomes are superior in reducing fungal burden in various organs including the brain and in prolonging animal survival.

## OBSERVATION

Cryptococcal meningitis has an exceptionally high global mortality rate of 60%, even with antifungal therapy (1). Inhalation of *Cryptococcus neoformans* cells is universal (2–4), and it typically leads to clearance or dormancy in the lungs of immunocompetent individuals. However, in immunocompromised people such as organ-transplant recipients and AIDS patients, the fungus reactivates and disseminates (5–8). After extrapulmonary dissemination, the fungus can invade all organs, with a propensity for the brain, causing fatal cryptococcal meningitis. Due to the severity of the disease, the lack of vaccines, and the limited success of current antifungal therapy, the World Health Organization (WHO) lists *C. neoformans* as a critical fungal pathogen. The treatment recommended by the WHO is a single dose of AmB-LLs (e.g., AmBisome) at 10 mg/kg coupled with either flucytosine or fluconazole for 7 days (9, 10). Using such a high dose of AmB-LLs can pose a challenge due to AmB toxicity issues that require hospitalization and close monitoring. Moreover, even with the recommended therapy, the mortality rate at 10 weeks is still ~25% (9).

To address this challenge, our group has developed DectiSomes, a targeted drug delivery system that directs antifungals to the fungal cell wall and extracellular polysaccharide matrix (11–13). We have focused primarily on AmB-loaded DectiSomes, as AmB is fungicidal and resistance to this drug is extremely rare. In addition, AmB is essential for successful cryptococcal treatment. By using DectiSomes, we expected to see increased drug efficacy without the need for a higher drug concentration, while maintaining low toxicity. Indeed, our previous study found that DectiSomes (Dec2-AmB-LLs) of the regular size (~100 nm), relative to the untargeted AmB-LLs (~100 nm, AmBisome), are modestly more effective against pulmonary and systemic cryptococcosis (14). Here, we tested our hypothesis that DectiSomes of a smaller size would penetrate the brain and other organs better and would further increase antifungal efficacy.

**Fig 1:**
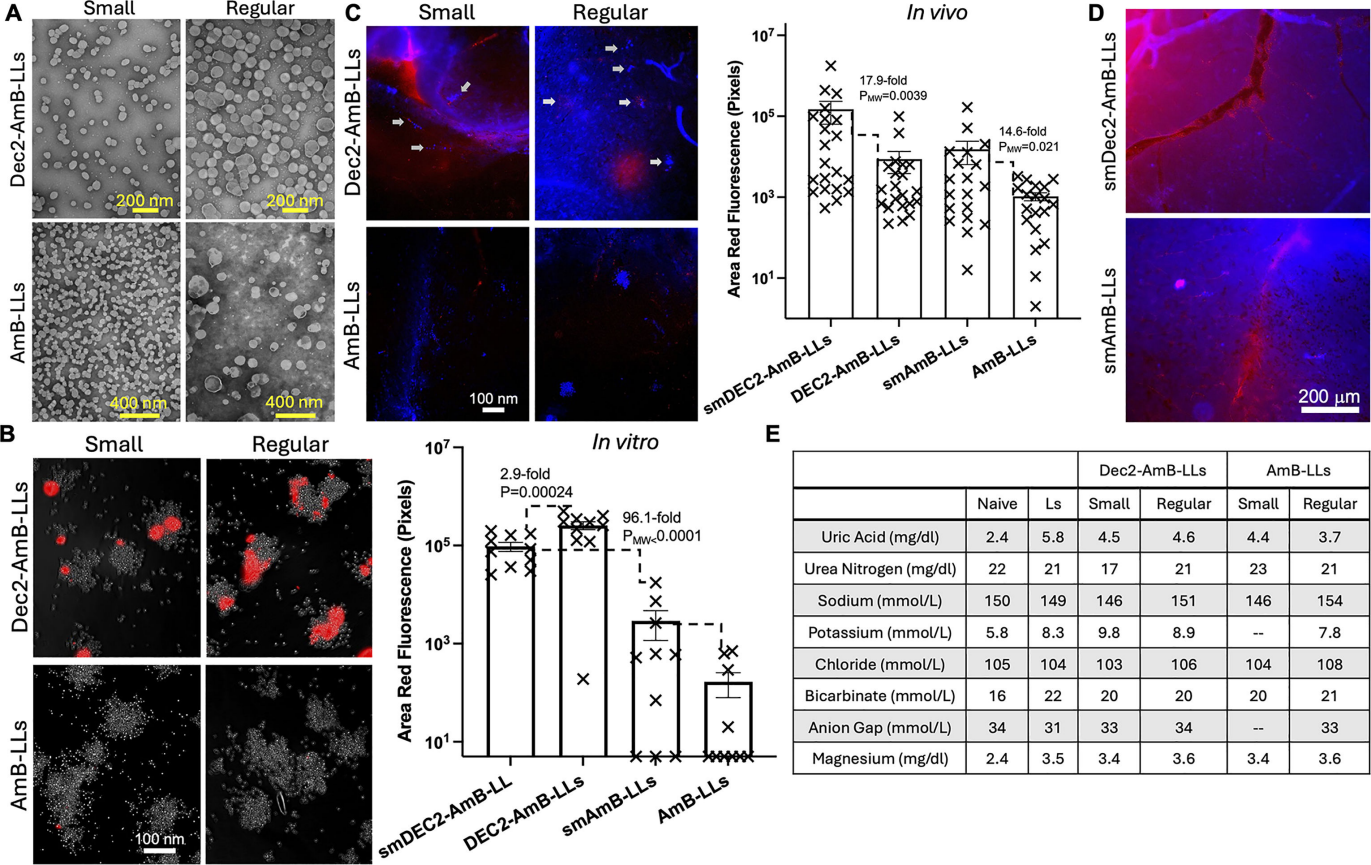
(A) Electron microscopy images of Dec2-AmB-LLs and AmB-LLs of the regular or the small size. (B) *In vitro* binding assay of regular and small Dec2-AmB-LLs and AmB-LLs labeled with rhodamine binding to cryptococcal cells. Quantification of the fluorescent signal intensity is shown in the graph to the right. (C) *In vivo* binding assay of regular and small Dec2-AmB-LLs and AmB-LLs labeled with rhodamine binding to cryptococcal cells stained with calcofluor white. Gray arrows pointing to the clusters of cryptococcal cells in the brain tissue. Quantification of the fluorescent signal intensity is shown in the graph top the right. (D) Accumulation of the rhodamine-labeled small liposomes in the vesicular tissue of the brain. (E) Kidney chemistry indexes using serum of mice treated with different types of liposomes.

To that end, we generated smaller DectiSomes through a membrane filtration method (Table S1). Based on electron microscopic images, our regular AmB-LLs were 92 nm in diameter in average, small AmB-LLs were 54 nm, Dec2-AmB-LLs were 88 nm, and small Dec2-AmB-LLs were 51 nm in size (Fig. 1A). This result is consistent with the particle size measurement of regular and small liposomes by Malvern Zetasizer prior to the loading of AmB or dectins (Table S1). Based on electron microscope images, all the liposomes of different sizes, with or without loading of AmB or dectins, appeared nearly spherical.

As the major advantage of DectiSomes is targeted binding to the fungal pathogen, we first compared the binding affinity of regular and small Dectin-2-targeted liposomes with untargeted liposomes to *C. neoformans* cells *in vitro*. We first labeled the liposomes red with rhodamine B and then measured the fluorescence intensity of these liposomes after co-incubation with cryptococcal cells. The small AmB-LLs showed over 20-fold higher fluorescence intensity than the regular AmB-LLs. Both the regular and the small Dectin-2-targeted DectiSomes showed about 100-fold higher fluorescence intensity than untargeted AmB-LLs (Fig. 1B). The differences between the binding by small Dec2-AmB-LL and the regular Dec2-AmB-LLs were relatively small (Fig. 1B). To test if regular and small DectiSomes have better binding to cryptococcal cells in host tissues, we then tested liposomal association of cryptococcal cells *in vivo*. Here, we infected mice intravenously with 5 × 10^6^ CFUs of cryptococcal cells and administered the four types of liposomes intravenously at 24 hours post-infection. We also included empty liposomes (Ls) as a negative control. At 24 hours post-treatment (48 hours post-infection), we sacrificed the mice, dissected the brain, hand sectioned the brain through the coronal plane, and stained topically with 10 µM calcofluor white for 10 minutes to reveal fungal cells before imaging. We found both the small AmB-LLs and small Dec2-AmB-LLs associated with fungal cells in statistically significant 10-fold higher amounts than their regular-sized counterparts, with small Dec2-AmB-LLs at the highest levels (Fig. 1C). We also noticed that the small liposomes accumulated in the brain’s blood vesicles (Fig. 1D). Our results suggest that the small liposomes penetrate the brain better. It is reasonable to speculate that higher vascular concentrations of liposomal AmB contributed to more efficient penetration of the blood brain barrier and more effective treatment of cryptococcal meningoencephalitis.

AmB is known to cause severe nephrotoxicity and electrolyte imbalance, although liposomal formulation mitigates such issues. Still high doses of AmB (e.g., 10, 15, or 20 mg/kg) have been required for liposomal formulations to be effective (15). Here, low doses of AmB were used (2.5 and 5 mg/kg). To test the impact of these liposomes on kidney function, we administered mice with empty Ls, the small- and the regular-size AmB-LLs, and the small- and the regular-size Dec2-AmB-LLs at the dose of 5 mg/kg of AmB intravenously. Sera from these mice were collected at day four post-treatment and were assayed for the levels of uric acid, urea nitrogen, sodium, potassium, chloride, bicarbonate, anion gap, creatinine, and magnesium. We found that mice treated with liposomes loaded with AmB at this dose had similar levels of all the indexes as those treated with empty liposomes or naïve mice without any treatment (Fig. 1E).

**Fig 2:**
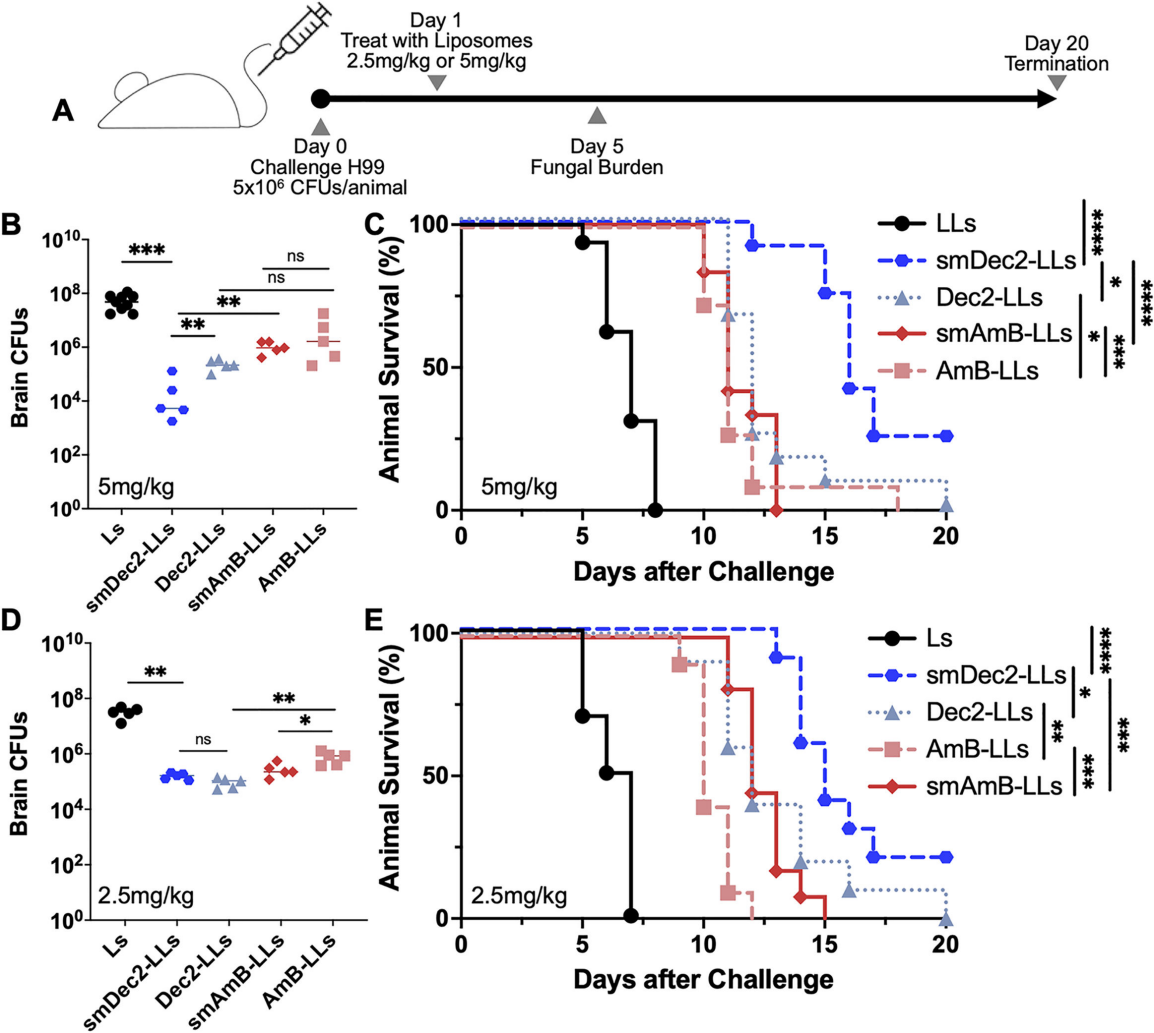
(A) Timeline of the treatment regimen for the animal experiment for panels B–E. (B) Quantification of CFUs of cryptococcal cells in the brain tissue at DPI 5 of mice treated with different liposomes at the AmB dose of 5 mg/kg (*n* = 10 mice per group). Nonparametric Mann-Whitney test was used for the statistical analysis. (C) Animal survival rate was plotted against days post-infection for each group of mice (Ls, smDec2-AmB-LLs, Dec2-AmB-LLs, AmB-LLs, and smAmB-LLs). The dose of AmB was 5 mg/kg. Ten mice were used for each group. Log rank test was used for the statistical analysis. (D) CFUs of cryptococcal cells in the brain tissue at DPI 5 of mice treated with different liposomes with AmB dose of 2.5 mg/kg. Ten mice per group were used in the experiment. Nonparametric Mann-Whitney test was used for the statistical analysis. (E) Animal survival rate was plotted against days post-infection for each group of mice (Ls, smDec2-AmB-LLs, Dec2-AmB-LLs, AmB-LLs, and smAmB-LLs). The dose of AmB was 2.5 mg/kg. Log rank test was used for the statistical analysis. ns: not significant; *P < 0.05, **P < 0.01, ***P < 0.001, and ****P < 0.0001

We then tested the efficacy of small and regular DectiSomes and AmB-LLs against *Cryptococcus* in a systemic infection model. Mice were infected intravenously with the clinical isolate H99 at 5 × 10^6^ CFUs/animal to initiate systemic cryptococcosis. In this model, the brain rapidly attains the highest fungal burden of any organ, and cryptococcal meningitis kills the mice within 10 days. At 24 hours post-infection, the infected mice were treated intravenously with empty liposomes lacking AmB (Ls) or the four types of liposomes delivering 5 mg/kg of AmB (Fig. 2A). On day 5 post-infection (DPI 5), mice were euthanized to examine the brain fungal burden. The median fungal burden for the Ls was 4.8 × 10^7^ CFUs, for AmB-LLs 3.33 × 10^5^ CFUs, for Dec2-AmB-LLs 1.51 × 10^5^ CFUs, for small AmB-LLs 5.45 × 10^5^ CFUs, and for small Dec2-AmB-LLs 5.32 × 10^3^ CFUs (Fig. 2B). Thus, all liposomes loaded with AmB reduced fungal burden in the brain, with small Dec2-AmB-LLs being the most effective. To examine if the reduced fungal burden translates to prolonged survival, we repeated the experiment and monitored animal survival. As expected, mice treated with Ls had a median survival of 7 days (Fig. 2C). All liposomes loaded with AmB prolonged the animal survival. Remarkably, mice treated with the small Dec2-AmB-LLs had a statistically significant longest median survival of 16 days, relative to the other treatments (Fig. 2C).

To determine if the superiority of small Dec2-AmB-LLs persists at a lower dose of AmB, we repeated the same brain fungal burden and survival experiments with half of the dose of AmB (2.5 mg/kg) (Fig. 2A). We tested fungal burden at DPI 5, when enough of the negative control-infected mice still survived. At this lower dose, all four types of liposomes loaded with AmB still drastically reduced brain fungal burden with the median for the Ls being 3.2 × 10^7^ CFUs, regular AmB-LLs 8.5 × 10^5^ CFUs, regular Dec2-AmB-LLs 1.07 × 10^5^ CFUs, small AmB-LLs 2.25 × 10^5^ CFUs, and small Dec2-AmB-LLs 1.65 × 10^5^ CFUs (Fig. 2D). Therefore, at this low dose, small AmB-LLs, regular Dec2-AmB-LLs, and small Dec2-AmB-LLs were all better than regular AmB-LLs. In terms of survival, mice treated with Ls had a median survival of 6.5 days, similar to the earlier experiment. All liposomes loaded with AmB at this lower 2.5 mg/kg dose significantly prolonged animal survival. Mice treated with small Dec2-AmB-LLs delivering 2.5 mg/kg had a median survival of 15 days, significantly longer than any other treatment (Fig. 2E). In short, small DectiSomes delivering either 2.5 mg/kg or 5 mg/kg of AmB drastically extended the lives of mice with cryptococcal meningitis.

These results demonstrate that the performance of DectiSomes can be considerably improved with optimization of liposome size. Small Dectin-2-targeted AmB-loaded DectiSomes provided the superior treatment of systemic cryptococcosis.

### Strains and growth conditions

*Cryptococcus* clinical isolate H99 was used in all experiments. H99 was stored in 15% glycerol at −80°C. Prior to use, it was streaked out fresh on yeast peptone D-glucose (YPD) medium incubated at 30°C.

### Preparation of DectiSomes

The 100-nm regular-sized DectiSomes were from FormuMax Sci. Inc. (Cat. F10203) composed of 1,2-distearoyl-sn-glycero-3-phosphocholine (DSPC) (18:0): cholesterol (CHOL): 1,2-distearoyl-sn-glycero-3-phosphoethanolamine-Poly(ethylene glycol) (DSPE-mPEG2000), 53:47:5 mole ratio (CAS#, 816-94-4, 57-88-5, and 147867-65-0). The new 50-nm liposomes were specially formulated and prepared for this project by FormuMax. They were composed of 1-palmitoyl-2-oleoyl-sn-*glycero*-3-phosphocholine (POPC) (16:0/18:1):CHOL:DSPE-mPEG2000, 58:40:2 mole ratio (CAS#, 26853-31-6, 57-88-5, and 147867-65-0). An ethanolic lipid solution with the POPC lipid mix was preheated to 60^o^–65°C and hydrated with nine volumes of phosphate-buffered saline (PBS) (pre-heated to 65°C) followed by incubation at 65°C for 1 hour. The suspension was extruded sequentially through 100-nm, 80-nm, and 50-nm pore size membranes. Their z-average size and volume-weighted mean sizes were estimated by light scattering on a Malvern ZetaSizer (Table S1). The 100-nm and 50-nm liposomes contain 5 mol% and 2% DSPE-mPEG-2000, respectively, relative to moles of liposomal lipid. We loaded 50 nm and 100 nm AmB-LLs with amphotericin B at 11 mol%, and the loading efficiency was determined using a subtractive method as described previously for 100 nm liposomes (11, 12). Dectin-2 and rhodamine were loaded at 1 and 2 mol%, respectively (11, 12).

### *In vitro* liposome binding assays

H99 cells were inoculated at 1 × 10^4^ cells/mL/well in a 24-well plate in Roswell Park Memorial Institute Medium (RPMI) and grown until achieving 60% to 80% confluence. Cells were washed once in PBS, fixed in 4% formalin in PBS for 1 hour, washed thrice in PBS, and blocked with liposome binding buffer (LDB2). The wells were then treated with the regular and small liposomes, delivering 1 µg of Dectin-2 per 100 μL of LDB2 (1:100 [wt/vol]) for 1 hour and an equivalent amount of AmB. Cells were washed thrice with LDB2 prior to inverted imaging.

### Brain tissue liposome binding assay

At DPI 1, mice were administered with 100 µL of rhodamine-labeled liposomes intravenously. Mice were euthanized at DPI 2. The brains were dissected, hand sectioned in the coronal plane with a razor blade, and stained with calcofluor white (10 µM) for 10 minutes prior to imaging. Microscope images were taken with a 10× lens (N.A. 0.35) using brightfield (PH1 condenser), red fluorescence using TxRED filters (Ex560/Em640) for viewing liposomes, or 4′,6-diamidino-2-phenylindole (DAPI) filters (Ex380/Em460) for viewing cryptococcal cells. When examining liposomes in the brain, tissue sections were viewed top down under a coverslip at 10× magnification. The CellProfiler subroutine, AreaPipe, was used to analyze the pixel areas of fluorescence.

### Microscopy

Transmission electron microscopy analysis of particles negatively stained with phosphotungstic acid (PTA)-stained was performed on a JEOL JEM1011 instrument with images taken at 100 kV or 200 kV following a previously published staining protocol (16). Light microscopy was performed on a RVSF1000/REVOLVE R4 microscope (VWR International, LLC). See Table S1.

### Murine models of cryptococcosis

#### Infection and treatment

Female CD-1 mice were purchased from the Charles River laboratories. Prior to infection, mice were acclimated to the UGA central animal facility. An initial inoculum of 10^6^ cells per mL of *Cryptococcus neoformans* strain H99 was cultured in 3 mL of liquid YPD medium shaking at 220 rpm at 30°C for 15 hours. Cells were washed three times with sterile saline and adjusted to 5 × 10^6^ cells/CFUs. Ls and AmB-loaded liposomes including AmB-LLs, Dec2-AmB-LLs, and Dec3-AmB-LLs were adjusted to either 2.5 mg/kg or 5 mg/kg of AmB and prepared as described previously (11, 12, 14). Mice sedated with isoflurane were immediately inoculated intravenously via the retro-orbital route with 100 µL of fungal cell suspension for infection or 100 µL of liposomes for treatment as we described previously (17–19). Mice were euthanized for fungal burden (DPI 5) or terminated when they reached the clinical endpoints for survival.

### Fungal burden analysis

At DPI 5, brains were dissected from euthanized mice, homogenized in 2 mL of cold PBS using an IKA-T18 homogenizer, serially diluted (10×), and plated onto yeast nitrogen base agar medium (17–19). After 2 days of incubation at 30°C, the plates were counted for CFUs.

### Statistical analysis

The CellProfiler AreaPipe output data for the area of red fluorescent liposome binding were collected in a .csv format file for their initial examination. Quantitative data were managed in Excel (v. 16.69) or GraphPad Prism 9 (v. 9.5.0). Non-parametric quantification of samples was estimated using the Mann-Whitney U test given as *P*_MW_. For survival, the log rank test was performed to determine the *P* values and indicated as ranges in the figure legend.

## Data Availability

All of the data supporting this study are presented herein.

## SUPPLEMENTAL MATERIAL

Table S1 mbio.02507-24-s0001.docx Characteristics of small liposomes composed of POPC/CHOL/mPEG2000-DSPE.

ASM does not own the copyrights to Supplemental Material that may be linked to, or accessed through, an article. The authors have granted ASM a non-exclusive, world-wide license to publish the Supplemental Material files. Please contact the corresponding author directly for reuse.
