# Supplementary material for: Small but mighty: targeted antifungal liposomes of a smaller size are superior in treating cryptococcal meningitis
Source: mBio. 2024 Nov 18;15(12):e02507-24. doi: 10.1128/mbio.02507-24 (PMC11633377; doi:10.1128/mbio.02507-24)
Supplement: Table S1 — Characteristics of small liposomes composed of POPC/CHOL/mPEG2000-DSPE. [file mbio.02507-24-s0001.docx]

**Table S1**. Characteristics of small liposomes composed of POPC/CHOL/mPEG2000-DSPE

| **Composition** | | | | | | | |
| --- | --- | --- | --- | --- | --- | --- | --- |
|  | Molecular weight | | % mol/mol | | mmol | mg | |
| POPC | 760.1 | | 58 | | 0.174 | 132.26 | |
| CHOL | 386.65 | | 40 | | 0.12 | 46.40 | |
| mPEG2000-DSPE | 2790.5 | | 2 | | 0.006 | 16.74 | |
| total |  | | 100 | | 0.3 | 195.40 | |
| **Particle features over time** | | | | | | | |
| Sample | Time point | Z-average (nm) | | Mean size  (vol-weighted nm) | | | PDI |
| Sample 1 | T = 0 | 68.03±0.5368 | | 56.19±2.93 | | | 0.171 |
|  | T = 2 wks | 67.68±0.4149 | | 58.33±1.70 | | | 0.175 |
|  | T = 8 wks | 68.12±0.8205 | | 58.29±3.92 | | | 0.157 |
| Sample 2 | T = 0 | 61.68±0.6214 | | 54.12±1.85 | | | 0.139 |
|  | T = 4 wks | 62.13±0.943 | | 55.35±1.95 | | | 0.114 |
|  | T = 17 wks | 68.31±0.1976 | | 55.59±0.37 | | | 0.155 |
| Sample 2 | T = 0 | 72.71±0.27 | | 58.67±4.73 | | | 0.193 |
|  | T = 10 wks | 71.04±0.4172 | | 58.32±3.11 | | | 0.182 |

**Note:** The liposomes were sterile filtered through 0.2 μm filters and stored in refrigerator (2-8°C). Particle sizes were measured using the same instrument (Malvern Zetasizer). The z-average and volume-weighed mean size and the polydispersity index (PDI) numbers were recorded as shown in the table. The liposomes were stable over the monitoring periods as indicated in the table.
